# Low population Japanese encephalitis virus (JEV) seroprevalence in Udayapur district, Nepal, three years after a JE vaccination programme: A case for further catch up campaigns?

**DOI:** 10.1371/journal.pntd.0007269

**Published:** 2019-04-15

**Authors:** Lance Turtle, Hannah E. Brindle, W. William Schluter, Brian Faragher, Ajit Rayamajhi, Rajendra Bohara, Santosh Gurung, Geeta Shakya, Sutee Yoksan, Sameer Dixit, Rajesh Rajbhandari, Bimal Paudel, Shailaja Adhikari, Tom Solomon, Mike J. Griffiths

**Affiliations:** 1 Institute of Infection and Global Health and National Institute for Health Research Health Protection Research Unit in Emerging and Zoonotic Infections, University of Liverpool, United Kingdom; 2 Royal Liverpool and Broadgreen University Hospitals, members of Liverpool Health Partners, Liverpool, United Kingdom; 3 Oxford University Clinical Research Unit, Ha Noi, Viet Nam; 4 World Health Organization, Kathmandu, Nepal; 5 Liverpool School of Tropical Medicine, Liverpool, United Kingdom; 6 Kanti Children’s Hospital, Maharajgunj, Kathmandu, Nepal; 7 National Academy of Medical Sciences, Kathmandu, Nepal; 8 National Public Health Laboratory, Teku, Kathmandu, Nepal; 9 Institute of Molecular Biosciences, Mahidol University and Translational Research Unit, Chulabhorn Research Institute, Bangkok, Thailand; 10 Center for Molecular Dynamics Nepal, Thapathali, Kathmandu, Nepal; 11 Walton Centre NHS Foundation Trust, member of Liverpool Health Partners, Liverpool, United Kingdom; 12 Alder Hey Children’s NHS Trust, member of Liverpool Health Partners, Liverpool, United Kingdom; University of Washington, UNITED STATES

## Abstract

The live attenuated Japanese encephalitis (JE) vaccine SA14-14-2 has been used in Nepal for catch-up campaigns and is now included in the routine immunisation schedule. Previous studies have shown good vaccine efficacy after one dose in districts with a high incidence of JE. The first well-documented dengue outbreak occurred in Nepal in 2006 with ongoing cases now thought to be secondary to migration from India. Previous infection with dengue virus (DENV) partially protects against JE and might also influence serum neutralising antibody titres against JEV. This study aimed to determine whether serum anti-JEV neutralisation titres are: 1. maintained over time since vaccination, 2. vary with historic local JE incidence, and 3. are associated with DENV neutralising antibody levels. We conducted a cross-sectional study in three districts of Nepal: Banke, Rupandehi and Udayapur. Udayapur district had been vaccinated against JE most recently (2009), but had been the focus of only one campaign, compared with two in Banke and three in Rupandehi. Participants answered a short questionnaire and serum was assayed for anti-JEV and anti-DENV IgM and IgG (by ELISA) and 50% plaque reduction neutralisation titres (PRNT_50_) against JEV and DENV serotypes 1–4. A titre of ≥1:10 was considered seropositive to the respective virus. JEV neutralising antibody seroprevalence (PRNT_50_ ≥ 1:10) was 81% in Banke and Rupandehi, but only 41% in Udayapur, despite this district being vaccinated more recently. Sensitivity of ELISA for both anti-JEV and anti-DENV antibodies was low compared with PRNT_50._ DENV neutralising antibody correlated with the JEV PRNT_50_ ≥1:10, though the effect was modest. IgM (indicating recent infection) against both viruses was detected in a small number of participants. We also show that DENV IgM is present in Nepali subjects who have not travelled to India, suggesting that DENV may have become established in Nepal. We therefore propose that further JE vaccine campaigns should be considered in Udayapur district, and similar areas that have had fewer vaccination campaigns.

## Author summary

In Nepal, immunisation using a live attenuated vaccine is given against Japanese encephalitis (JE), caused by the mosquito-transmitted JE virus (JEV). JE immunisation has taken place via catch-up campaigns and is now part of the routine immunisation programme. Although previous studies have shown good vaccine efficacy in areas where there is a lot of natural exposure to the virus (high endemicity), it is suggested that the efficacy may wane in areas where transmission is lower. Dengue virus (DENV) belongs to the same family and genus as JEV. Previous infection with DENV may also influence the immune response to JEV. Therefore, we conducted a cross-sectional study in Nepal to measure immunity to JE, in districts of differing historic JE incidence, and time from JE vaccination. This showed that neutralising antibody to JEV was found more frequently in districts which had been the subject of more vaccination campaigns, rather than in the most recently vaccinated district. In addition, we cannot rule out a role for natural exposure to JEV in maintaining higher antibody levels. Additionally, the study showed that previous exposure to DENV was positively associated with an immune response to JEV, though this effect was modest. We conclude that there is a need to consider further JE vaccine catch up campaigns in some areas especially given that we could detect JEV IgM, indicating ongoing transmission. We show that ELISA yielded many false negative results for exposure to JEV or vaccination, when compared with neutralising antibody. We also identified some individuals during the course of the study with DENV IgM in their blood, but with no history of travel to India. This suggests that DENV may have become established in some areas of Nepal.

## Introduction

Japanese encephalitis virus (JEV), an arthropod-borne virus belonging to the genus *Flavivirus* of the family *Flaviviridae*, is transmitted by *Culex* mosquitoes, with birds and pigs acting as natural reservoirs and amplifying hosts. Humans are an accidental dead-end host [1]. JEV is enzootic in many parts of rural South and Southeast Asia where it is a common cause of viral encephalitis, particularly in children [1–4]. Less than 1% of those infected develop symptoms [1]. Disease usually occurs in childhood or in adults who live in areas of low enzootic circulation of JEV and may be non-immune [2]. JE begins as an undifferentiated febrile illness followed in some by the development of seizures, altered sensorium or other neurological signs [5, 6]. Occasionally, acute flaccid paralysis and Parkinsonism occur [7, 8]. The case fatality rate is 20%-30% [2]; and neuropsychiatric disability is seen in up to 50% of survivors [9–12].

JE vaccines were first developed in the 1950s, and several types have been used [13]. JEV SA14-14-2 is an attenuated vaccine strain derived from JEV SA14, which was isolated from mosquitoes in China in the late 1940s. JEV SA14-14-2 was developed in China and licensed in 1988. JE was first seen in Nepal in 1978 [14] but vaccination against JE using SA14-14-2 was not introduced in the country until July 1999 when 224,000 doses of the vaccine were administered to children aged 1–15 years (as 0.5 ml intramuscular administration of single dose) in the Bardiya, Banke and Kailali districts [15]; however, reported vaccination coverage between the districts was variable (Bardiya 83%, Banke 41% and Kailali 22%)[16]. Between 2005 and 2010, 62 of the 75 districts in Nepal reported cases of JE, mostly those at lower altitude, thought to be because at higher altitudes there is less mosquito activity [17]. From 2006 to 2011, 31 high and moderate JE risk districts were included in a phased catch-up immunisation campaign; some districts vaccinated everyone in the population older than 1 year of age whereas others vaccinated only those aged 1–15 years [16, 18]. In addition, three doses of inactivated JE vaccine were given between 2000 and 2001 to children aged 6 months to 10 years living in the districts of Banke, Rupandehi, Kailali, Dang, and Kanchanpur [19]. In 2000, a single dose of SA14-14-2 vaccine was given to 98 children aged 1–15 years in Chitwan as part of a vaccine efficacy study [20]. Currently, JEV SA14-14-2 is given to children aged 12–23 months living in 31 high JE risk districts in Nepal as part of the national immunisation schedule introduced in a step-wise manner starting with 22 districts in 2009 and then expanded to 31 districts by 2012 [16]. Given the combination of catch-up campaigns and routine immunisation it is possible that a proportion of the population were vaccinated more than once. Despite cases of JE in Nepal still occurring particularly in the summer months [21], vaccination has resulted in a 78% reduction in the number of JE cases nationally [22].

Anti-JEV antibodies that develop after SA14-14-2 vaccination wane over time [23]. Whether further doses are required, however remains unclear, as clinical protection against JE may still occur despite low neutralising antibody titres [23, 24]. The efficacy of JE vaccine SA14-14-2 in a study in 2000 in Bardiya and Banke districts (higher JE incidence) was 98.5% after one year [25] falling very slightly to 96.2% after five years [26]. However, In Chitwan, an area of lower JE endemicity, neutralising antibodies against JEV persisted in only 63.8% of subjects 5 years after vaccination in 2000 [20] and a study from India showed that vaccine efficacy reduced from 87% in those vaccinated less than one year ago to 66% in those vaccinated a year or longer ago [27]. It has been shown that in older populations, after the antibodies wane following vaccination, they may rise again after natural infection [23]. Given that JE is seen more frequently in children in areas where the virus is not newly established [1, 28] it is therefore possible that natural infection may play a role in protection and a single dose of the vaccine may be sufficient in areas where maintenance of the antibody response, or of immunity, occurs due to high rates of natural infection.

Dengue virus (DENV) is a flavivirus closely related to JEV that is transmitted by the *Aedes aegypti and Aedes albopictus* mosquitoes [29]. Dengue has only recently been documented in Nepal with the earliest case seen in 2006. Initially dengue cases were thought to be secondary to the movement of people from India, but more lately indigenous cases have occurred. In 2010 there was an outbreak in Chitwan and Rupandehi districts resulting in 917 cases. In 2012–2013 there were 77 laboratory confirmed cases [30]. Spatially, there have been cases of both JE and dengue documented in a number of districts [17]. There is immunological cross-reactivity between flaviviruses but the clinical effect of this is variable. Previous infection with DENV reduces the severity of subsequent JE [31, 32]. Vaccination with an inactivated JE vaccine might reduce the severity of dengue fever modestly [33]. Conversely, in a more recent study in Thailand, anti-JEV antibodies predisposed to symptomatic dengue fever, as opposed to clinically silent infection [34]. Despite the variance in clinical findings, given that JEV and DENV have been found in many of the same districts in Nepal [17], induction or maintenance of JE immunity by DENV infection is a theoretical possibility, leading to higher JEV seroprevalence, but less disease.

The Nepali districts of Banke, Rupandehi and Udayapur are all JE enzootic. Banke reported 16.7 JE cases per 100,000 in 2004–6, tenfold higher than Rupandehi and Udayapur (1.6/100,000 in 2004–6) [18]. All three districts have been targeted by JE vaccination campaigns: Banke in 1999; Banke and Rupandehi in 2000–1 and 2006; and Udayapur in 2009. These JE vaccination campaigns were undertaken in all ages, with immunisation coverage rates reported to be 86% and over in all age groups [16]. Subsequently, the vaccination of children was introduced into the national programme for immunisation in all three districts in 2009 with vaccination coverage of 97% in Rupandehi, 76% in Banke and 71% in Udayapur [16]. Banke saw a reduction in the number of cases of JE, from ninety-four in 2005 to three in 2012. This compared with a reduction from nineteen cases in 2005 to zero in 2012 in Rupandehi, and from two cases to zero in Udayapur over the same period [16].

Udayapur is at higher altitude than Banke and Rupandehi, although the incidence of JE in 2004–6 in Udayapur and Rupandehi was the same, despite the difference in altitude, indicating an environment that can sustain JEV transmission in Udayapur.

**Fig 1:**
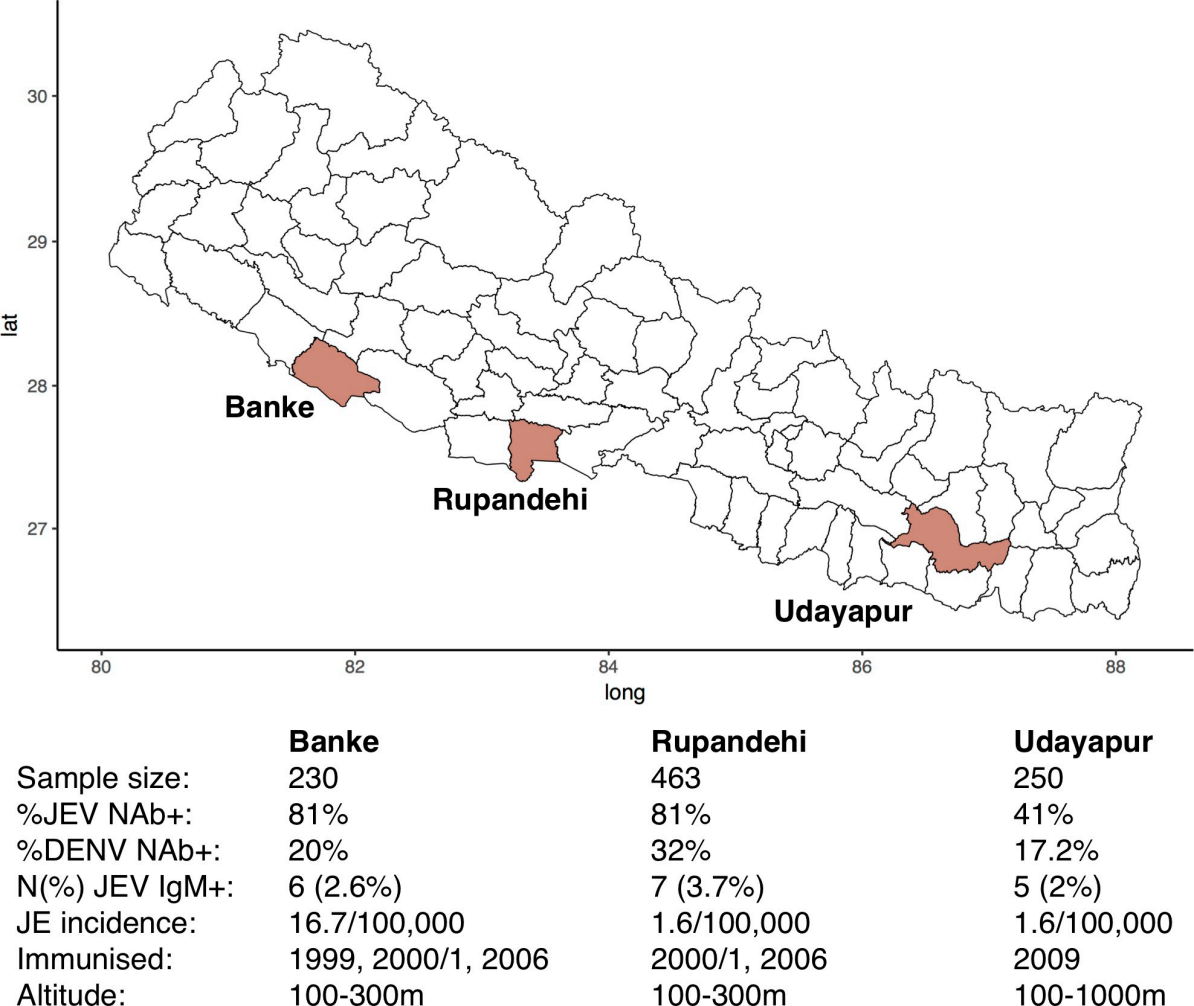
The brown coloured areas represent the districts included in the study with the dates of vaccination campaigns and the level of endemicity (cases/100,000 population in 2004–6). The percentage of participants seropositive for JEV and DENV are also shown, along with the number (%) JEV IgM positive, and the altitude range of the district.

The existence of three districts of Nepal with varying JE incidence, which have been the target of single dose JE vaccination campaigns at different times in all ages regardless of JEV transmission intensity, provided an opportunity to compare the seroprevalence of JEV according to time from vaccination and level of historical JEV transmission (Fig 1). Moreover, there may be benefit from further JE vaccination catch-up campaigns in low JE incidence districts. This study was designed to answer the following questions:

1. Is anti-JEV neutralising antibody maintained over time (by comparing residents of districts vaccinated 3 years apart but with the same historic level of endemicity)?
  *Hypothesis: anti-JEV-antibody sero-prevalence would be higher among subjects from Udayapur compared with Rupandehi due to more recent vaccination campaigns*.
2. Do antibody levels vary with natural exposure to JEV (by comparing historic high incidence districts with lower incidence districts vaccinated at the same time)?
  *Hypothesis: anti-JEV-antibody sero-prevalence would be higher among subjects from Banke compared with Rupandehi due to a higher natural exposure to JEV*.
3. Are anti-DENV and anti-JEV antibodies associated with each other?
  *Hypothesis: anti-JEV and anti-DENV neutralisation titres would be positively associated due to immunological cross-reactivity where there is co-circulation of the viruses*.

## Methods

A sero-prevalence survey was conducted across the three districts of Banke, Rupandehi and Udayapur in Nepal (Fig 1). Banke and Rupandehi were sampled during October–November, and Udayapur in December 2012.

Fieldwork was performed by research assistants employed by the Center for Molecular Dynamics, Nepal (CMDN). A representative random sample was selected in each of the three study districts using community-based stratified multi-stage cluster methodology as follows:

■ Districts in Nepal are divided into Village Development Committees (VDCs) which are further divided into wards. Wards and VDCs were selected in each study district using the random number generator in Microsoft Excel.
■ Households were then chosen at random within each ward using a method from the Expanded Programme for Immunisation coverage survey which is applied when household lists are unavailable in rural areas. A bottle was spun in the centre of the ward and the number of houses counted whilst walking in a straight line to the edge of the ward in the direction indicated by the bottle—a random sample of these houses was then selected for inclusion in the survey [35].
■ The number of participants selected per district was proportional to the population size of the district. Consequently, a higher number of subjects were recruited from Rupandehi.

Based on the average number of households visited during vaccination and surveillance campaigns, it was estimated that eight households per day could be surveyed. Sampling of 600 households was therefore deemed adequate within the time frame allocated for recruitment which was up to a maximum of six months. Accounting for 10% refusal rate, this would leave 540 households. It was estimated that on average, two adults per household would be recruited giving a total number of participants of 1080. The number of households per district was calculated based on the population proportional to sample size. Assuming that 50% of the total population sampled were JEV seropositive, using the R package ‘epiR’ and the function epi.clustersize, nine clusters (VDCs) would give a 95% certainty of being within 13% of the true population.

Residents aged 19 years and over and who were present within the household at the time of recruitment were invited to participate in the study. This age group was selected for reasons of practicality, as drawing blood samples from well children in this setting can be challenging and a high rate of non-consent was assumed. In addition, as the vaccination campaigns included adults as well as children, sampling adults would give adequate insight into population level immunity, and would exclude sampling people too young to have been included in the campaigns. Written consent was obtained from participants after they had read or, in the case of those unable to read and write, had listened to a researcher reading out, the detailed description of the survey procedures contained in the participant information sheet. Those who were unable or unwilling to complete this consent process were excluded.

Participants who consented to take part in the survey answered questions relating to their demographics, travel history (including family travel history), and recent history of fever within the past year or symptoms suggestive of JE or dengue fever using a simple questionnaire (Supp. File 1). A 5ml blood sample was collected for serum separation. All samples were collected in the period September–December 2012. No cases of dengue virus were found in any of the three study districts in 2012–2013 [30].

Anti-JEV IgM antibodies in serum were measured using the InBios JE *detect*^TM^ IgM capture ELISA (sensitivity 99.2%, specificity 56.1% compared with the AFRIMS in house ELISA [36]; sensitivity 57%, specificity 95.4% compared with the CDC diagnostic algorithm including neutralising antibody [37]). Anti-DENV IgM antibodies in serum were measured using the InBios Dengue *detect*^TM^ IgM capture ELISA (sensitivity 88.7%, specificity 93.1% [38]). IgG antibodies were measured using the In Bios JE and DENV *detect*^TM^ IgG ELISAs, at the National Public Health Laboratory, Teku, Nepal. ELISAs were carried out according to the manufacturer’s instructions. For IgM ELISAs, test samples were incubated in wells coated with anti-human IgM antibody, followed by incubation with recombinant viral antigen or a control normal cell antigen (NCA), and anti-viral detection antibody. For IgG ELISAs, test samples were incubated with recombinant viral antigen (captured onto the plate via a monoclonal antibody), and NCA. The assay results are then expressed as a ratio of viral antigen to NCA, referred to as the immune status ratio (ISR). An ISR of >5 was considered positive, and an ISR of 2–5 equivocal. The DENV In Bios ELISA uses recombinant antigens of all four serotypes of DENV. Fifty percent plaque reduction neutralisation titres (PRNT_50_) against JEV (strain P3) and DENV serotypes 1–4 (Dengue 1: 16007, Dengue 2: 16681, Dengue 3: 16562, Dengue 4: C036/6) were measured at Mahidol University, Bangkok, Thailand using the method of Russell et al. (1967) [39]. Three serum ten-fold dilutions were used starting at 1:10 except in the case of two samples where there was minimal serum (<0.4ml) so the first dilution was 1:20.

### Ethics approval

Ethical approval was obtained from Liverpool School of Tropical Medicine (LSTM) (12.19RS) and the Nepal Health Research Council (Reg no. 5/2012). The study was conducted according to the Declaration of Helsinki. All participants gave written informed consent.

### Statistical analysis

Data were analysed using STATA version 13 and R statistical software (www.r-project.org). For the dichotomous outcome measures, differences between the three study districts were evaluated using Fisher’s exact test and the z test to calculate the difference between proportions; adjustment was then made for important covariates using negative binomial regression with robust standard error estimates to take into account clustering effects at the VDC level. For PRNT_50_ < 1:10, values recorded as zero were imputed as exact figures were not available. Antibody levels were transformed to geometric mean titres to remove positive skewness and differences were compared using the Wilcoxon signed rank test, differences between ISR were also compared using the Wilcoxon signed rank test.

## Results

**Fig 2:**
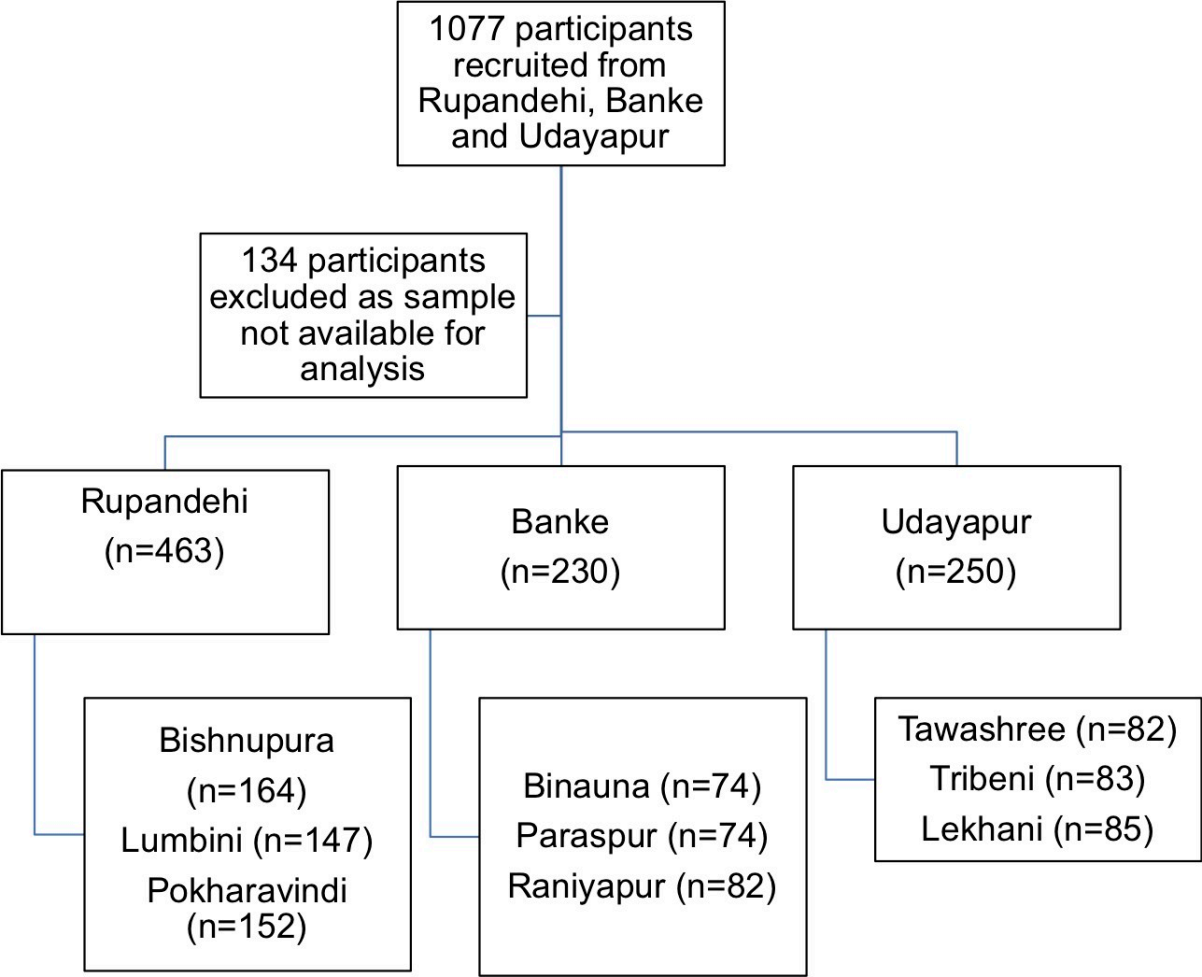
Recruitment process of participants in the three Nepali districts of Rupandehi, Banke and Udayapur. In each district, recruitment was conducted in three Village Development Committees (VDC, bottom boxes), selected at random (see methods). Recruitment numbers in each VDC are indicated.

A total of 1077 participants were recruited, of whom 943 provided fully completed consent forms and serum samples for antibody measurement (Fig 2).

The demographic characteristics of these participants are summarised in Table 1. More females (59.8%; 95%CI 56.6–62.9%) than males (40.2%; 95%CI 37.1–43.4%) were recruited into the study with this difference being consistent across all districts. Overall, 23.2% (95%CI 20.6–26.1%) and 26.1% of participants (95%CI 23.3–29.0%) had visited India within the last year or had a family member who had. A higher proportion of respondents or their family members from Banke (26.1%, 95%CI 20.5–32.3% and 33.0%, 95%CI 27.0–39.5% respectively) and Rupandehi (32%, 95%CI 27.7–36.4% and 26.8%, 95%CI 22.8–31.1% respectively) had visited India within the last year compared with Udayapur (4.4%, 95%CI 2.2–7.7% and 18.4%, 95%CI 13.8–23.8% respectively). Sixty-one percent (95%CI 58.3–64.6%) of all respondents were farmers. Of those from Rupandehi, a higher proportion were farmers compared with the other districts. Twenty-five percent (95%CI 22.5–28.1%) of respondents had anti-DENV PRNT_50_ of ≥1:10; a higher proportion of those living in Rupandehi had an anti-DENV PRNT_50_ of ≥1:10 compared to the other districts (Table 1). The median age of respondents was 35 years and duration of residency, 27 years. More young than older participants were enrolled in all three districts, with a similar age profile in each district (S1 Fig).

**Table 1:** Demographic characteristics of participants overall and by district: N (%; 95% CI). *median (range).

		All participants	Banke	Rupandehi	Udayapur
Sample size		943	230	463	250
**Gender**^**†**^	Male	379 (40.2; 37.1–43.4)	93 (40.4; 34.0–47.1)	192 (41.6; 37.0–46.2)	94 (37.6; 31.6–43.9)
	Female	563 (59.8; 56.6–62.9)	137 (59.6; 52.9–66.0)	270 (58.4; 53.8–63.0)	156 (62.4; 56.1–68.4)
**Travel to India within** **last year**	Yes	219 (23.2; 20.6–26.1)	60 (26.1; 20.5–32.3)	148 (32.0; 27.7–36.4)	11 (4.4;2.2–7.7) 239
	No	724 (76.8; 73.9–79.4)	170 (73.9; 67.7–79.5)	315 (68.0; 63.6–72.3)	(95.6; 92.3–97.8)
**Family member travelled to India in last year**	Yes	246 (26.1; 23.3–29.0)	76 (33.0; 27.0–39.5)	124 (26.8; 22.8–31.1)	46 (18.4; 13.8–23.8)
	No	697 (73.9; 71.0–76.7)	154 (67.0; 60.5–73.0)	339 (73.2; 68.9–77.2)	204 (81.6; 76.2–86.2)
Farming occupation**	Yes	577 (61.4; 58.3–64.6)	129 (56.6; 49.9–63.1)	336 (72.7; 68.4–76.7)	112 (45.0; 38.7–51.4)
	No	362 (38.6; 35.4–41.7)	99 (43.4; 36.9–50.1)	126 (27.3; 23.3–31.6)	137 (55.0; 48.6–61.3)
**Anti-JEV PRNT**_**50**_≥**1:10**	Yes	666 (70.6; 67.6–73.5)	187 (81.3; 75.5–86.0)	375 (81.0; 77.1–84.4)	102 (40.8; 35.5–48.0)
	No	277 (29.4; 26.5–32.4)	43 (18.7; 14.0–24.5)	88 (19; 15.6–22.9)	148 (59.2; 52.0–64.5)
**Anti-DENV PRNT**_**50**_≥**1:10**	Yes	238 (25.2; 22.5–28.1)	46 (20.0; 15.0–25.8)	149 (32.2; 27.9–36.6)	43 (17.2; 12.7–22.5)
	No	705 (74.8; 71.9–77.5)	184 (80.0; 74.2–85.0)	314 (67.8; 63.4–72.1)	207 (82.8; 77.5–87.3)
**Median age of participants (years)**		*35 (19–85)	*35 (19–72)	*35 (19–85)	*33.5 (19–81)
**Median duration of residency (years)**		*27 (0.08–85)	*30 (1–70)	*27 (0.58–85)	*23.5 (0.08–74)

† not recorded for 1 participant in Rupandehi

** not recorded for 4 participants, 1 in Rupandehi, 1 in Udayapur and 2 in Banke

**Fig 3:**
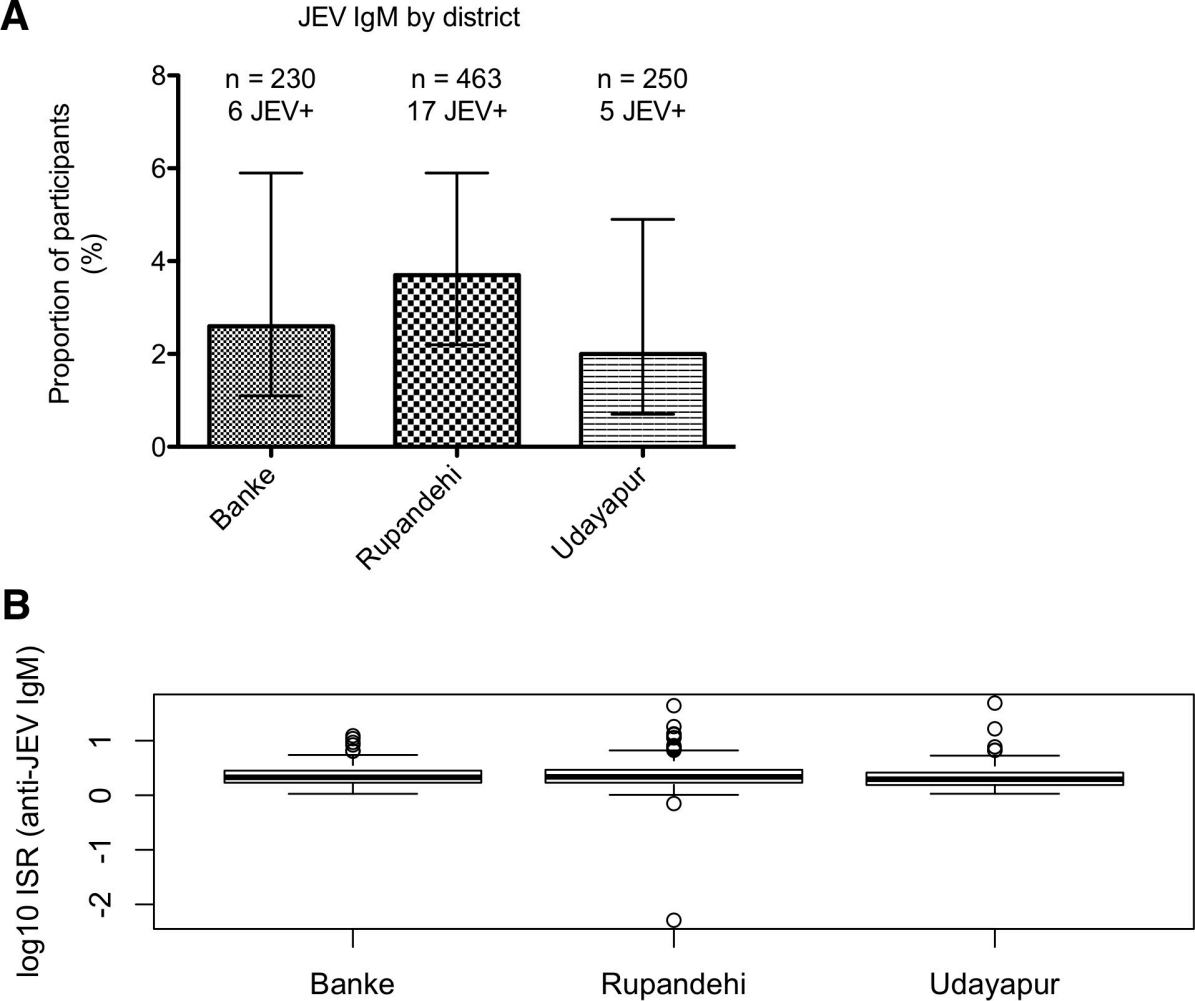
JEV IgM ELISA results by district. A, percentage and numbers of participants with anti-JEV IgM in each district, with 95% confidence intervals. B, JEV IgM ELISA values by district of the anti-JEV IgM log_10_ immune status ratio (ISR, the ratio of optical density readings obtained for the JEV and normal cell antigens for each sample). Thick bars represent the median, boxes the interquartile range, error bars 1.5x the IQR, and outliers are shown as open circles.

All 943 serum samples were assessed for the presence of JEV and DENV antibody by neutralisation assay (PRNT_50_), and IgM and IgG using InBios JE and DENV detect diagnostic ELISAs. Twenty-eight samples had anti-JEV IgM detected by ELISA (3.0%; 95% CI 2.1–4.3%), of which nine were JEV IgG negative, 17 equivocal and 2 were IgG positive, indicating recent infection and ongoing enzootic circulation of JEV. Six IgM positive subjects were from Banke, 17 from Rupandehi and five from Udayapur; the proportion of samples tested that were JEV IgM positive was similar across all three districts (Fig 3A). Ninety-six samples were anti-JEV IgG positive (10.2%; 95% CI 8.4–12.3%), 20 from Banke, 58 from Rupandehi and 18 from Udayapur. IgM ELISA results for each district are shown in Fig 3, expressed as the ratio of OD obtained for JEV antigen over normal cell antigen for the same sample (Immune Status Ratio, ISR). The median anti-JEV IgM log_10_ ISRs for Banke and Rupandhi were 0.33 and 0.34 respectively, corresponding to ISR values of 2.14 and 2.19. The value for Udayapur was 0.29 (ISR 1.95), significantly lower (p = 0.007). Seven samples had anti-DENV IgM (0.7%; 95% CI 0.4–1.5%) of whom one had been to India within the last year.

**Fig 4:**
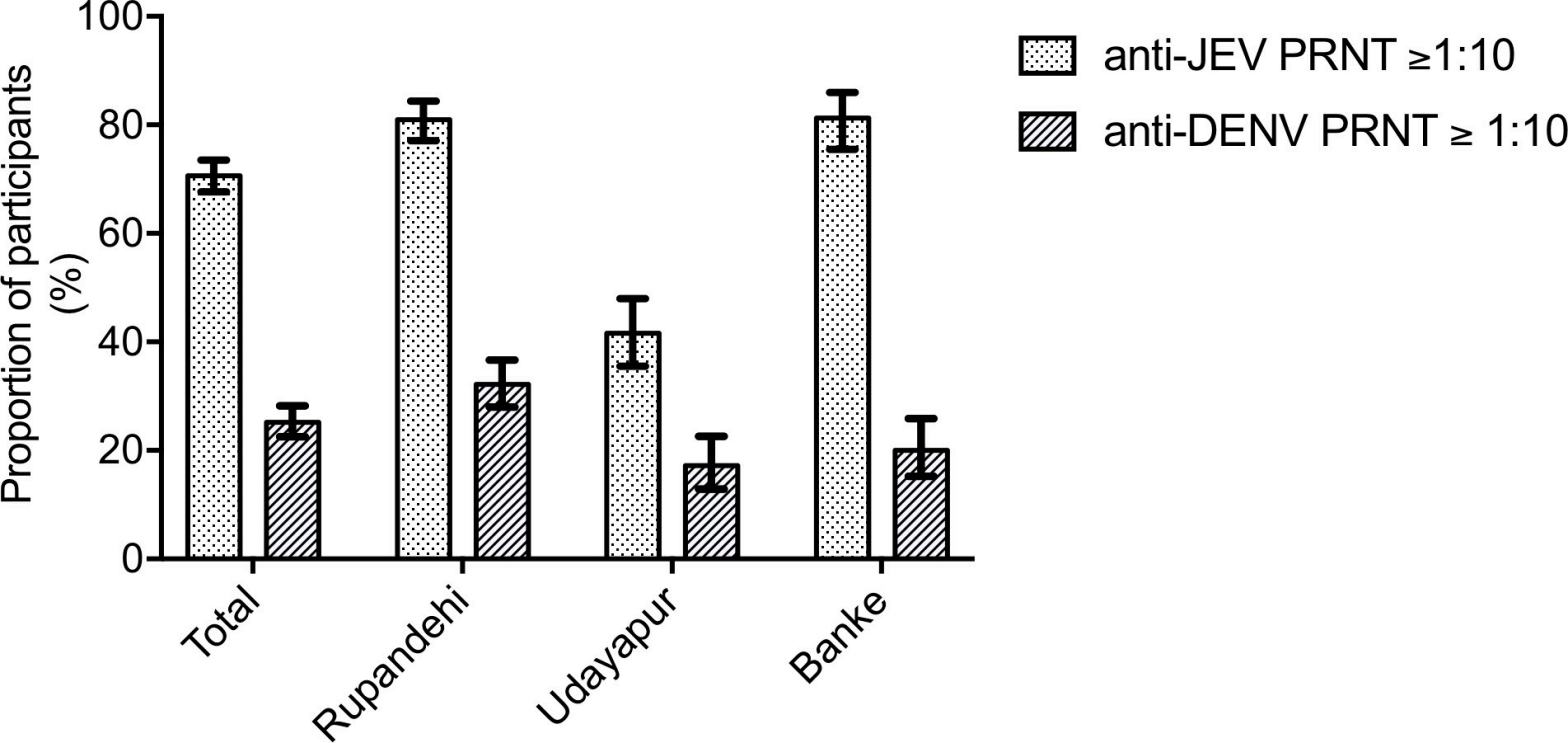
Seroprevalence of JEV and DENV antibody by 50% plaque reduction neutralisation titre (PRNT_50_). Data are the percentage of participants with PRNT_50_ ≥1:10, error bars indicate the 95% confidence interval.

The proportion of participants that had anti-JEV neutralising antibody (PRNT_50_ ≥1:10) differed across the study districts: seroprevalence was 81% in Banke (95% CI 75.5–86%) and Rupandehi (95% CI 77.1–84.4%), whereas it was significantly lower, 41%, in Udayapur (95% CI 35.4–48.0), the district with low historical JE incidence but the most recent vaccine coverage (2009) [Fisher’s exact test for comparison between Udayapur, and Banke and Rupandehi combined, p<0.001] (Fig 4). In this population, therefore, ELISA was insensitive for the detection of both anti-JEV and anti-DENV antibody compared with PRNT_50_, with sensitivity 18% (95% CI 15–21%) for JEV and 54% (95% CI 48–61%) for DENV (Table 2). Combining equivocal and positive results improved the sensitivity (59 (95% CI 55–63%) for JEV, 73% (95% CI 67–79%) for DENV), but still left a significant proportion of samples testing negative. For the JEV IgG ELISA alone, more suited to detection of immune memory, the performance of the assay was influenced by the presence of DENV neutralising antibody. For example, the sensitivity of the assay in DENV NAb negative subjects was only 47% (95% CI 42–52%) but the sensitivity in DENV NAb positive subjects was 71% (95% CI 65–77%), with a corresponding drop in specificity (Table 2). This indicates that the JEV IgG ELISA cross-reacts to some extent with DENV antibody.

**Table 2:** Sensitivity, specify, positive and negative predictive values of anti-JEV and anti-DENV ELISAs using PRNT as the reference test. Data are shown for IgG and IgM combined (as both can contribute to neutralisation), and for the IgG ELISA alone (for use in sero-surveys).

	JEV IgG or IgM positive (95% CI) n = 120	DENV IgG or IgM positive (95% CI) n = 179	JEV IgG or IgM positive or equivocal (95%CI) n = 421	DENV IgG or IgM positive or equivocal (95% CI) n = 370	JEV IgG positive or equivocal, All (95% CI) n = 379	JEV IgG positive or equivocal, DENV NAb neg (95% CI) n = 226	JEV IgG positive or equivocal, DENV NAb pos (95% CI) n = 153
**Sensitivity**	0.18	0.54	0.59	0.73	0.54	0.47	0.71
	(0.15–0.21)	(0.48–0.61)	(0.55–0.63)	(0.67–0.79)	(0.48–0.6)	(0.42–0.52)	(0.65–0.77)
**Specificity**	1.00	0.93	0.91	0.72	0.94	0.97	0.71
	(0.99–1.00)	(0.91–0.95)	(0.87–0.94)	(0.69–0.75)	(0.88–0.99)	(0.95–0.99)	(0.57–0.85)
**Positive predictive value**	1.00	0.72	0.94	0.47	0.95	0.97	0.93
	(0.97–1.00)	(0.65–0.79)	(0.91–0.96)	(0.42–0.52)	(0.90–1.00)	(0.95–0.99)	(0.89–0.97)
**Negative predictive value**	0.34	0.86	0.48	0.89	0.46	0.48	0.32
	(0.30–0.37)	(0.83–0.88)	(0.44–0.52)	(0.86–0.91)	(0.33–0.58)	(0.44–0.53)	(0.22–0.42)

**Fig 5:**
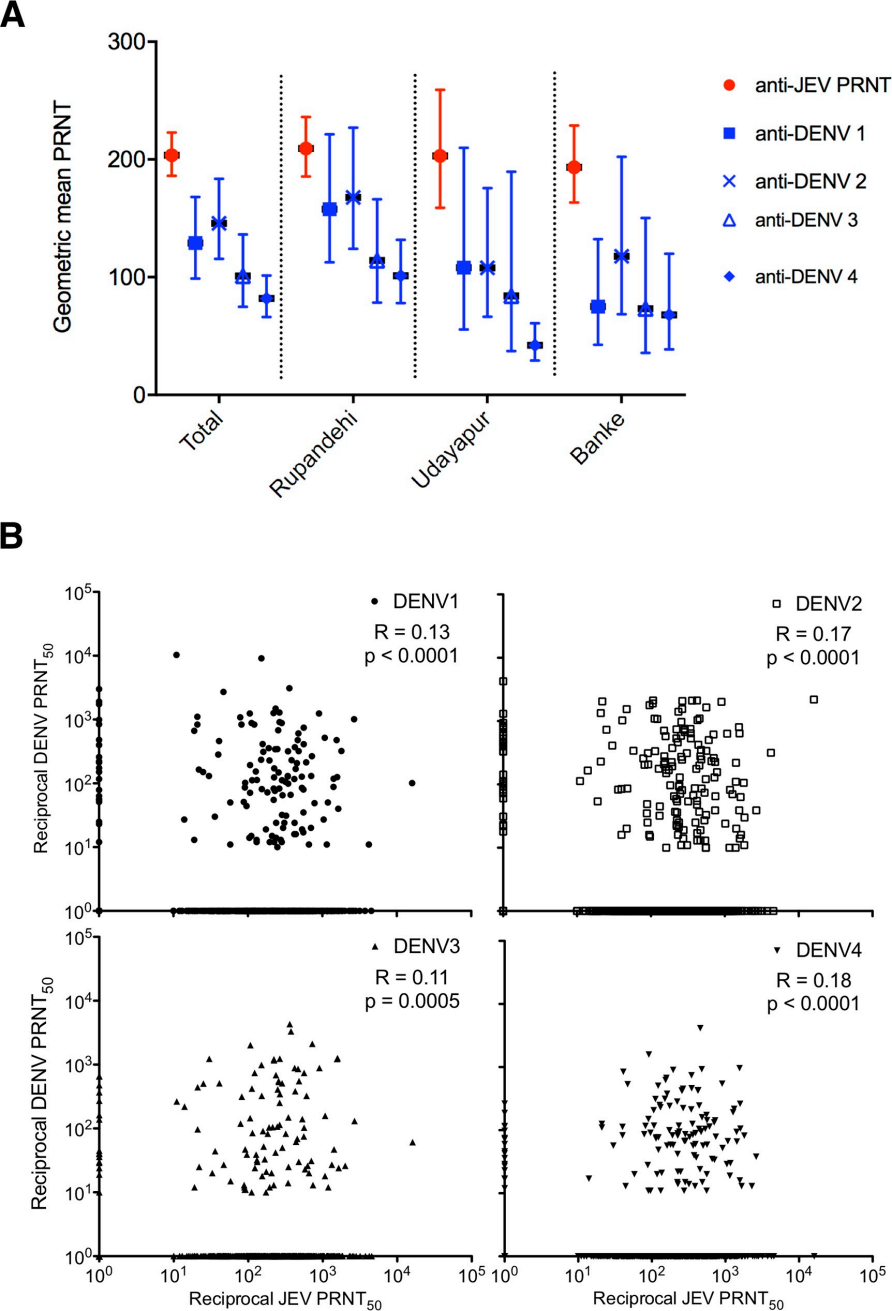
A geometric mean (error bar: 95% confidence interval) of JEV and DENV serotypes 1–4 neutralising titres in the three districts. B Relationship between DENV and JEV neutralising titres (PRNT_50_) for each of the four DENV serotypes. Samples with PRNT_50_ ≤ 1:10 have been assigned a value of 1 for the purpose of plotting these data. Spearman’s R and p values are shown for each DENV serotype.

Seroprevalence of anti-DENV antibody measured by PRNT_50_ ≥1:10 was 25.2% (95% CI 22.5–28.1), or 238 individuals sero-positive (Fig 4). As was the case for JEV, this proportion differed significantly across the study districts: DENV seroprevalence in Banke was 20% (95% CI15.0–25.8), 32.2% in Rupandehi (95% CI 27.9–36.6) and 17.2% in Udayapur (95%12.7–22.5) [Fisher’s exact test between Rupandehi, and Banke and Udayapur combined, p<0.001]. Evidence of past infection by DENV serotype 2 was detected with DENV serotype 2 antibody found most commonly in 18.6% of participants (95% CI 16.1–21.2) and at highest titre (Fig 5). Most subjects with DENV neutralising antibody had antibody to two or more flaviviruses (220 of 238), and although most had antibody to DENV multiple serotypes, a number of participants had neutralising antibody only to DENV2, or to DENV2 and JEV. Similar, though less frequent, examples of monotypic DENV neutralising antibody could be identified for serotypes 1 and 4 (S2 Fig). No convincing monotypic antibody responses were identified for DENV serotype 3. In keeping with the likely circulation of DENV2, antibody titres to this serotype were higher, though less than anti-JEV titres (Fig 5A). The magnitude of JEV and DENV neutralising antibody titres were also correlated, though modestly (Spearman’s R 0.11–0.18, Fig 5B).

The proportion of respondents with an anti-JEV PRNT_50_ ≥1:10 remained significantly lower in Udayapur than in both Banke and Rupandehi after adjustment (multivariate logistic regression) for age, gender, travel to India in previous year of self or a family member, working as a farmer and a level of anti-DENV PRNT_50_ ≥1:10 (Table 3). There was no significant difference between the proportion of respondents with an anti-JEV PRNT_50_ ≥1:10 in Banke and Rupandehi. Increasing age and a level of anti-DENV PRNT_50_ ≥1:10 were also identified as being (independently) associated with a level of JEV PRNT_50_ ≥1:10. JEV and DENV antibody titres were also correlated with each other, though the effect was modest (Spearman’s r 0.11–0.18, p < 0.0005 for all DENV serotypes, Fig 5).

**Table 3:** Factors independently associated with an anti-JEV PRNT_50_ ≥1:10 (n = 938).

		IRR	95% CI	p value
**Age (years)**	19–29	1		
	30–39	1.172	1.020–1.346	0.025
	40–49	1.304	1.129–1.507	<0.001
	50+	1.301	1.140–1.485	<0.001
**Gender**	Female	1		
	Male	1.013	0.901–1.139	0.826
**District of residence**	Banke	1		—
	Rupandehi	0.973	0.850–1.115	0.696
	Udayapur	0.519	0.311–0.866	0.012
**Travelled to India in** **previous year**	No	1		— 0.799
	Yes	0.989	0.908–1.077	
**Family member travelled to India in the previous year**	No	1		
	Yes	1.065	0.967–1.174	0.200
**Farming occupation**	No	1		
	Yes	0.986	0.902–1.078	0.754
**DENV PRNT**_**50**_ ≥**1:10**	No	1		
	Yes	1.183	1.043–1.342	0.009

IRR = incidence rate ratio CI = confidence interval

After a similar adjustment (multivariate logistic regression) for age, gender, travel to India in previous year of self or a family member and working as a farmer, the proportion of respondents with an anti-DENV PRNT_50_ ≥1:10 remained significantly lower in both Udayapur and Banke than in Rupandehi, the district with lower historical JE incidence and vaccinated earlier (2006). In addition, women were observed to be significantly less likely than men to have anti-DENV PRNT_50_ ≥1:10 (Table 4) however, significantly more males than females had travelled to India within the past year (31.7% (95% CI 27.1–36.7%) of males compared to 17.6% (95%CI 14.6–21% of females, p<0.001). There was no significant difference between the proportion of respondents with an anti-DENV PRNT_50_ ≥1:10 in Udayapur and Banke.

**Table 4:** Factors independently associated with an anti-DENV PRNT_50_ ≥1:10 (n = 938).

		IRR	95% CI	p value
**Age (years)**	19–29	1		
	30–39	0.998	0.743–1.340	0.988
	40–49	1.255	0.908–1.734	0.169
	50+	1.243	0.805–1.919	0.325
**Gender**	Female	1		
	Male	1.438	1.103–1.874	0.007
**District of residence**	Banke	1		
	Rupandehi	1.524	1.083–2.144	0.016
	Udayapur	0.940	0.596–1.482	0.790
**Travelled to India in** **previous year**	No	1		
	Yes	1.312	0.884–1.946	0.178
**Family member travelled to** **India in previous year**	No	1		
	Yes	0.947	0.694–1.293	0.732
**Farming occupation**	No	1		
	Yes	1.166	0.742–1.833	0.505

IRR = incidence rate ratio CI = confidence interval

## Discussion

The first objective of this study was to determine whether anti-JEV neutralising antibody levels are maintained over time in Nepal (by comparing residents of districts vaccinated 3 years apart) with the hypothesis that anti-JEV-antibody sero-prevalence would be higher among subjects from Udayapur compared with Rupandehi due to more recent vaccination campaigns. Secondly, we hypothesised that anti-JEV-antibody sero-prevalence would be higher among subjects from Banke compared with Rupandehi due to a higher natural exposure to JEV. In this study, the district targeted by the most recent vaccination campaign (Udayapur) had the lowest sero-prevalence of JEV neutralising antibody. Overall, approximately 70% of participants across the three districts surveyed had anti-JEV PRNT_50_ ≥1:10, but sero-prevalence was only 41% in Udayapur compared with 81% in Rupandehi and Banke. This difference is not accounted for by reported differences in vaccination coverage, but may be due to a number of factors.

Banke and Rupandehi have both participated in more vaccination campaigns than Udayapur (Banke has had three campaigns, Rupandehi two but Udayapur only one) so it is possible that some participants in these two districts had received multiple doses of the vaccine, contributing to the higher sero-positive rate seen in these districts. Previous work has also shown that the impact of vaccination on JE incidence was lower in moderate-risk hill districts compared to high-risk Terai districts (43 compared with 84% reduction in incidence) [40]. Other reasons do potentially exist for these differences, such as differences in vaccine coverage rates (for example vaccine coverage in Udayapur may have been lower than estimated), vaccine handling, or the target population, rather than the underlying level of natural exposure to JEV [40]. However, in a longer term follow up study, although the impact remained greatest in the high-risk Terai districts, JE incidence was 62% lower in moderate risk Terai districts, 69% lower in moderate risk hill districts, and 89% lower in high-risk Terai districts, narrowing the gap somewhat between the high and moderate risk districts, and suggesting a causal role for JE vaccination [22].

The geography and climate of Udayapur is distinct to Banke and Rupandehi. Only 34% of the Udayapur district is classified as being ‘lower tropical’ with an elevation of 0-300m). In contrast, 79% and 89% of Banke and Rupandehi districts respectively are classified lower tropical [41]. It is therefore possible that there is less transmission potential in Udayapur. Additionally, JEV may have circulated in Banke and Rupandehi for longer than Udayapur; Rupandehi was the first reported district in Nepal to see JE in 1978 and it is thought that the virus was brought into Nepal from neighbouring North India, which shares a border with both Banke and Rupandehi but not with Udayapur [17]. Repeated natural exposure to JEV may result in maintenance of neutralising antibody, and hence could be another explanation for our findings. Although historically Rupandehi and Udayapur had similar JE incidence, this may not have remained the case and more recent data are not available.

JEV IgM antibodies were found in participants living in all three districts with the highest proportion occurring in Rupandehi. However, the low numbers of IgM positive results in this study make it difficult to form conclusions about differences in recent JEV infections between the districts. We may have underestimated the number of recent infections in Udayapur, because this district was sampled slightly later than the other districts in December; in Nepal the JE season is August–September. This may also account for lower ELISA values in Udayapur. Despite this, these results indicate that JEV transmission is on-going in all three districts. Lastly, the occurrence of IgM antibodies may have been underestimated in Banke and Rupandehi as well, which were also sampled after the JE season (October and December), introducing the possibility that some participants had lost IgM after infection earlier in the same year.

Although the fewer number of JE vaccination campaigns in Udayapur may be sufficient to explain the difference in seroprevalence, we cannot completely rule out a role for less natural transmission of JEV between Udayapur and the other two districts. The most likely explanation may be that some combination of these two factors explains the difference. Because we have not directly measured JEV transmission in these districts, we cannot say for certain what the relative importance of each factor is. Nevertheless, given that JEV clearly still circulates in Udayapur (indicated by the presence of JEV IgM), consideration of repeated vaccination campaigns in low/moderate JE incidence areas such as this is warranted.

We also hypothesised anti-JEV and anti-DENV neutralising antibody titres would be positively correlated. We found a significant, though modest, correlation between PRNT_50_ ≥1:10 against DENV and JEV that remained after adjustment for differences between districts, a finding that is in keeping with previous studies, supporting our hypothesis [42]. Additionally, it was found that residents of Banke were significantly less likely to have detectable DENV neutralising antibody compared with those from Rupandehi. This is consistent with the occurrence of 150 cases of dengue fever in Rupandehi during the 2010 outbreak, whereas Banke saw none. Previously it has been thought that dengue fever in Nepal is mostly imported from India, but this study found no association between anti-DENV PRNT_50_ ≥1:10 and travel to India and only one out of seven with a positive DENV IgM who had crossed the border. This finding is consistent with the establishment of DENV (possibly serotype 2) in some districts of Nepal, at least temporarily, potentially informing the public health response to future dengue cases. Rupandehi had significantly higher DENV sero-prevalence than Udayapur, but Banke had only marginally higher DENV sero-prevalence. Combined with the modest association of DENV and JEV antibody, therefore, maintaining anti-JEV antibody by DENV infection is not a likely explanation for the difference in JEV sero-prevalance between districts.

We found that ELISA had a low sensitivity compared with PRNT for detection of anti-JEV antibody. However, PRNT is expensive, labour intensive and not suitable for many settings. Moreover, neutralisation assays can become unreliable at low serum dilations which might be necessary to test the immune response to SA14-14-2 over a longer time period, where dilutions of less than 1:10 may be needed as SA14-14-2 does not always elicit a high level of neutralising antibodies [43]. Therefore, from a public health perspective, JE vaccination campaigns should be directed in response to CSF JEV IgM positive cases rather than population serosurveys.

One limitation of this study is that there is no method for differentiation between antibody to JEV acquired by natural infection as opposed to vaccination. Although one study showed potential for a JEV NS1-specific indirect ELISA to differentiate between JE vaccination and past infection [44], this study was based on inactivated JE vaccine and live JE vaccine SA14-14-2 also contains NS1, which may induce antibody responses and therefore would be unable to definitively differentiate between the two. As it was not possible to determine how many vaccinations a participant had received, all were included in the main analysis even if they had moved to the study district after the vaccination campaign. This does not, however, affect our conclusions. Despite being unable to differentiate between the causes of acquisition of immunity to JEV, an understanding of the seroprevalence in the region is important to help guide immunisation campaigns, although, accurate identification of vaccination status would undoubtedly be beneficial. From an immunological perspective, it is not known whether it is simply the presence, or magnitude, of neutralising antibodies correlates with clinical protection against JEV. For example, amnestic antibody responses [45], or cellular immune responses [43], may also contribute and could be a marker of exposure even if antibody neutralisation is absent. Although we cannot differentiate a neutralising antibody negative but otherwise primed (by vaccination or exposure) individual from a completely naïve individual, it is nevertheless likely that there are more susceptible individuals in Udayapur than the other two districts under study. Finally, this study was conducted in adults, whereas JE is mostly seen in children. However, in this regard, our data likely represent an under-estimate of the susceptible population, which would only re-enforce our primary conclusion. Clearly, robust durable neutralising antibody responses have not developed in Udayapur province.

Future work could extend and improve upon our findings, including: inclusion of a paediatric cohort; running a longer prospective cohort study; more detailed immunological studies to determine more accurate markers of exposure or immunity beyond neutralising antibody alone. Finally, a large prospective study undertaken across areas of differing JE incidence could address the durability of immunity. However, such a study would be challenging and expensive to conduct.

Both JE and dengue are seasonal in Nepal with JE cases peaking in August/September [46] and outbreaks of dengue in 2009 and 2010 occurring in September–October and August–December respectively [17]. Therefore, although our study period overlapped with these months, it is possible that prevalence of IgM antibodies would have been under-estimated compared with earlier in the year. Additionally, vaccine coverage was estimated to be high and similar in all three districts, independent confirmation of this is lacking. Finally, as the study was conducted in 2012 we cannot account for more recent changes in JE epidemiology. For example, cases are now seen in districts with higher elevation and *Culex* mosquitoes are found at higher elevations possibly due to the warming effect of climate change [47]. However, this does not affect our primary conclusion, that consideration of further JE vaccination campaigns in Udayapur is warranted. Alternatively, strengthening or extending the current routine immunisation schedule could obviate the need for such campaigns, at least in the paediatric population.

Although it could be argued the reduction in JE incidence is lower in low/moderate risk compared to high risk districts following vaccination [22, 40], we nevertheless recommend consideration of further JE vaccination campaigns in areas of low/moderate JE incidence and in any district where JEV IgM is detected. However, the need for catch-up campaigns should be informed by data from outbreaks/reintroduction of the virus, and also by the extent of coverage of the routine immunisation program, across all age groups. If routine immunisation coverage were high enough, including in adults, catch up campaigns would no longer be required. Finally, our findings suggest that DENV has the potential to become established in Nepal, a finding of significant public health importance.

## Supporting information

S1 Dataset (XLSX)

S1 STROBE Checklist (DOCX)

S1 Fig Frequency distribution histogram of ages of participants in the three districts sampled. Histogram bins are 5 years wide, the x axis indicates the central value of each bin.
(PDF)

S2 Fig Evidence of specific DENV serotype circulation. (A) DENV neutralising antibody (NAb) titres in participants positive (PRNT50 ≥ 1:10) for only one DENV serotype. (B) DENV neutralising antibody titres in participants positive (PRNT50 ≥ 1:10) for only one DENV serotype, who are also JEV NAb negative. (C) JEV NAb titres in participants positive for one DENV serotype. Open diamonds = DENV1 NAb. Open squares = DENV2 NAb. Open triangles = DENV3 NAb. Closed inverted triangles = DENV4 NAb. Open circles = JEV NAb.
(PDF)
