# Supplementary material for: Low population Japanese encephalitis virus (JEV) seroprevalence in Udayapur district, Nepal, three years after a JE vaccination programme: A case for further catch up campaigns?
Source: PLoS Negl Trop Dis. 2019 Apr 15;13(4):e0007269. doi: 10.1371/journal.pntd.0007269 (PMC6483279; doi:10.1371/journal.pntd.0007269)
Supplement: S1 STROBE Checklist — (DOCX) [file pntd.0007269.s002.docx]

STROBE Statement—Checklist of items that should be included in reports of ***cross-sectional studies***

|  | Item No | Recommendation |  |  |  | Comment | Lines |  |  |
| --- | --- | --- | --- | --- | --- | --- | --- | --- | --- |
| **Title and abstract** | 1 | (*a*) Indicate the study’s design with a commonly used term in the title or the abstract |  |  |  | Included | Abstract para 2 |  |  |
|  |  | (*b*) Provide in the abstract an informative and balanced summary of what was done and what was found |  |  |  | Included | Abstract paras 2 & 3 |  |  |
| Introduction | | |  |  |  |  |  |  |  |
| Background/rationale | 2 | Explain the scientific background and rationale for the investigation being reported |  |  |  | Included | Introduction paras 2-5 |  |  |
| Objectives | 3 | State specific objectives, including any prespecified hypotheses |  |  |  | Included | Introduction para 6 |  |  |
| Methods | | |  |  |  |  |  |  |  |
| Study design | 4 | Present key elements of study design early in the paper |  |  |  | Included | Methods paras 1-3 |  |  |
| Setting | 5 | Describe the setting, locations, and relevant dates, including periods of recruitment, exposure, follow-up, and data collection |  |  |  | Included | Methods para 1 |  |  |
| Participants | 6 | (*a*) Give the eligibility criteria, and the sources and methods of selection of participants |  |  |  | Included | Methods paras 4 & 5 |  |  |
| Variables | 7 | Clearly define all outcomes, exposures, predictors, potential confounders, and effect modifiers. Give diagnostic criteria, if applicable |  |  |  | Included | Methods para 6 |  |  |
| Data sources/ measurement | 8* | For each variable of interest, give sources of data and details of methods of assessment (measurement). Describe comparability of assessment methods if there is more than one group |  |  |  | Included | Methods paras 6 & 7 |  |  |
| Bias | 9 | Describe any efforts to address potential sources of bias |  |  |  | Included | Methods para 2 |  |  |
| Study size | 10 | Explain how the study size was arrived at |  |  |  | Included | Methods para 3 |  |  |
| Quantitative variables | 11 | Explain how quantitative variables were handled in the analyses. If applicable, describe which groupings were chosen and why |  |  |  | Included | Methods para 8 |  |  |
| Statistical methods | 12 | (*a*) Describe all statistical methods, including those used to control for confounding |  |  |  | Included | Methods para 8 |  |  |
|  |  | (*b*) Describe any methods used to examine subgroups and interactions |  |  |  | NA |  |  |  |
|  |  | (*c*) Explain how missing data were addressed |  |  |  | Included (figure 2 – only those with samples available for analysis)  NA for questionnaires | Results, figure 2 |  |  |
|  |  | (*d*) If applicable, describe analytical methods taking account of sampling strategy |  |  |  | Included | Methods para 8 |  |  |
|  |  | (*e*) Describe any sensitivity analyses |  |  |  | Included | Results para 4 |  |  |
| Results | | |  |  |  |  |  |  |  |
| Participants | 13* | (a) Report numbers of individuals at each stage of study—eg numbers potentially eligible, examined for eligibility, confirmed eligible, included in the study, completing follow-up, and analysed |  |  |  | Included figure 2 (although number screened for eligibility unknown) | Results figure 2 |  |  |
|  |  | (b) Give reasons for non-participation at each stage |  |  |  | See above |  |  |  |
|  |  | (c) Consider use of a flow diagram |  |  |  | Included | Results figure 2 |  |  |
| Descriptive data | 14* | (a) Give characteristics of study participants (eg demographic, clinical, social) and information on exposures and potential confounders |  |  |  | Included | Results table 1 |  |  |
|  |  | (b) Indicate number of participants with missing data for each variable of interest |  |  |  | NA |  |  |  |
| Outcome data | 15* | Report numbers of outcome events or summary measures |  |  |  | Included | Results para 3 & 5 |  |  |
| Main results | 16 | (*a*) Give unadjusted estimates and, if applicable, confounder-adjusted estimates and their precision (eg, 95% confidence interval). Make clear which confounders were adjusted for and why they were included |  |  |  | Included  (confounder-adjusted only) | Results para 5 |  |  |
|  |  | (*b*) Report category boundaries when continuous variables were categorized |  |  |  | Included | Results tables 3 & 4 |  |  |
|  |  | (*c*) If relevant, consider translating estimates of relative risk into absolute risk for a meaningful time period |  |  |  | NA |  |  |  |
| Other analyses | 17 | Report other analyses done—eg analyses of subgroups and interactions, and sensitivity analyses |  |  |  | Included | Results para 5, tables 3 & 4 |  |  |
| Discussion | | |  |  |  |  |  |  |  |
| Key results | 18 | Summarise key results with reference to study objectives |  |  |  | Included | Discussion para 1 |  |  |
| Limitations | 19 | Discuss limitations of the study, taking into account sources of potential bias or imprecision. Discuss both direction and magnitude of any potential bias |  |  |  | Included | Discussion paras 8 & 9 |  |  |
| Interpretation | 20 | Give a cautious overall interpretation of results considering objectives, limitations, multiplicity of analyses, results from similar studies, and other relevant evidence |  |  |  | Included | Discussion para 2, 5-7, 9 |  |  |
| Generalisability | 21 | Discuss the generalisability (external validity) of the study results |  |  |  | Included | Discussion para 10 |  |  |
| Other information | | |  |  |  |  |  |  |  |
| Funding | 22 | Give the source of funding and the role of the funders for the present study and, if applicable, for the original study on which the present article is based |  |  |  | Included |  | NA |  |

*Give information separately for exposed and unexposed groups.

**Note:** An Explanation and Elaboration article discusses each checklist item and gives methodological background and published examples of transparent reporting. The STROBE checklist is best used in conjunction with this article (freely available on the Web sites of PLoS Medicine at http://www.plosmedicine.org/, Annals of Internal Medicine at http://www.annals.org/, and Epidemiology at http://www.epidem.com/). Information on the STROBE Initiative is available at www.strobe-statement.org.
