# Supplementary figures and images for: Low population Japanese encephalitis virus (JEV) seroprevalence in Udayapur district, Nepal, three years after a JE vaccination programme: A case for further catch up campaigns?
Source: PLoS Negl Trop Dis. 2019 Apr 15;13(4):e0007269. doi: 10.1371/journal.pntd.0007269 (PMC6483279; doi:10.1371/journal.pntd.0007269)

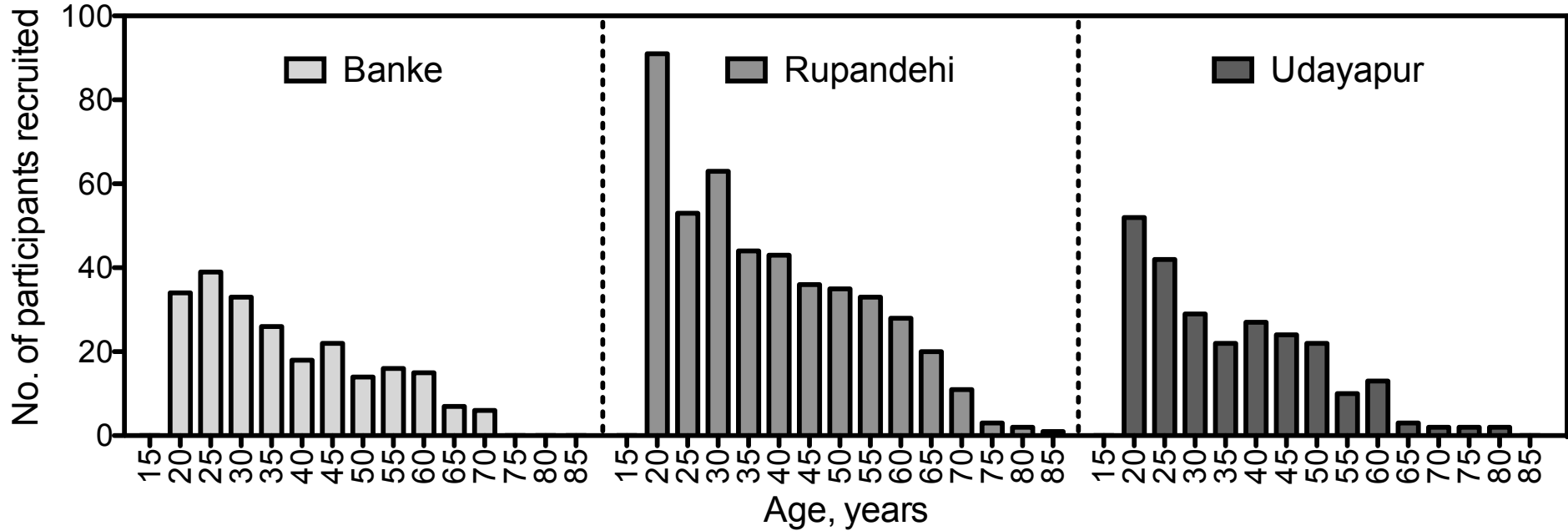

Supplement: S1 Fig — Histogram bins are 5 years wide, the x axis indicates the central value of each bin. (PDF) [file pntd.0007269.s003.pdf]

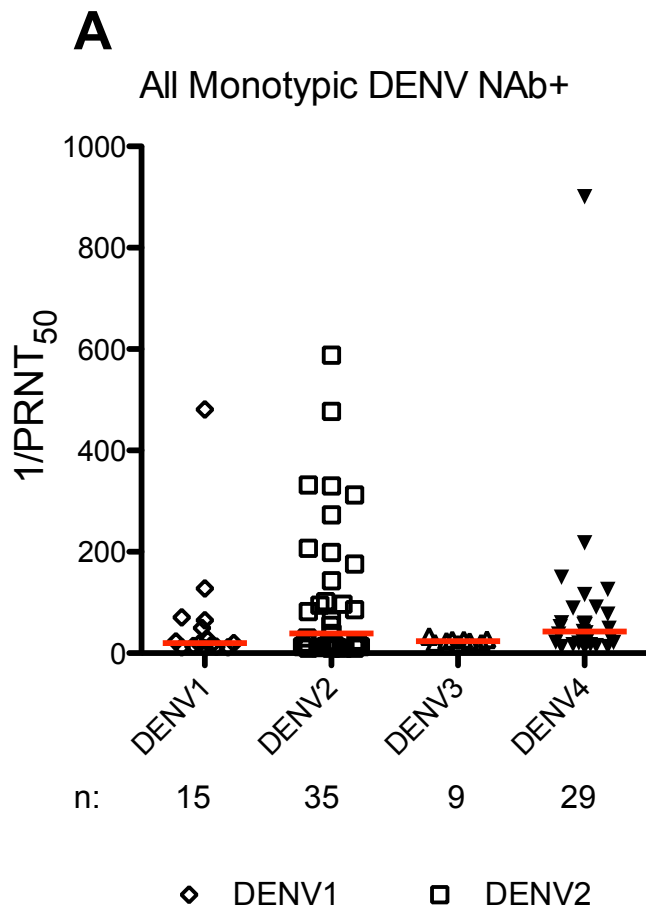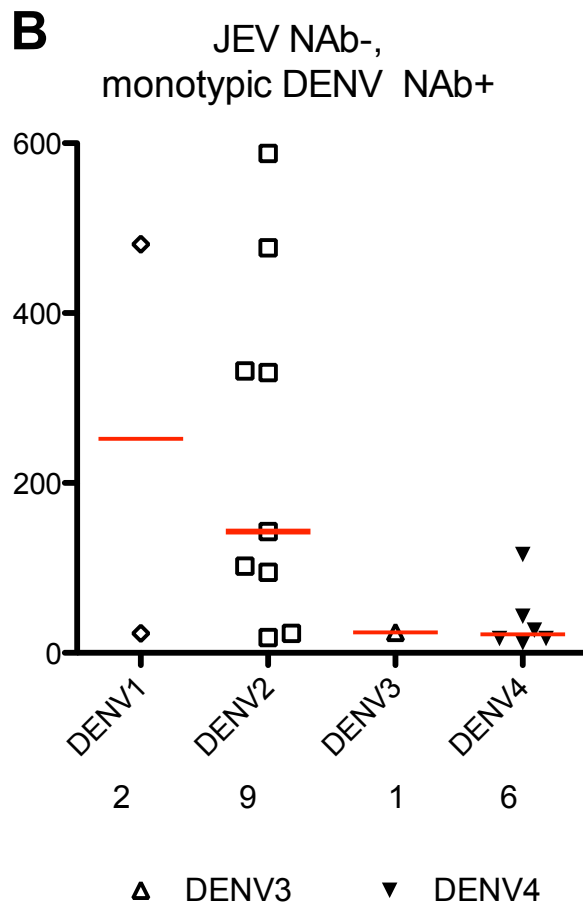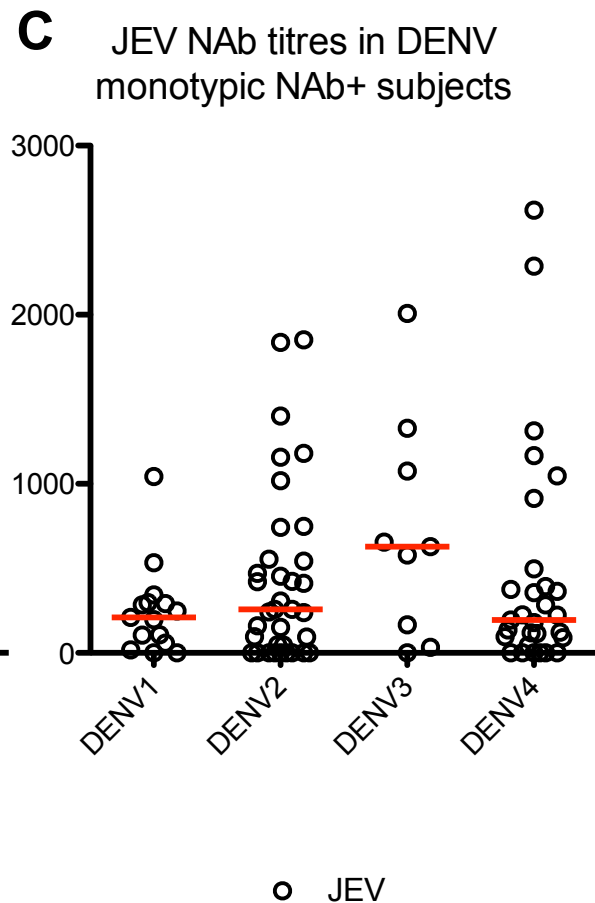

Supplement: S2 Fig — (A) DENV neutralising antibody (NAb) titres in participants positive (PRNT50 ≥ 1:10) for only one DENV serotype. (B) DENV neutralising antibody titres in participants positive (PRNT50 ≥ 1:10) for only one DENV serotype, who are also JEV NAb negative. (C) JEV NAb titres in participants positive for one DENV serotype. Open diamonds = DENV1 NAb. Open squares = DENV2 NAb. Open triangles = DENV3 NAb. Closed inverted triangles = DENV4 NAb. Open circles = JEV NAb. (PDF) [file pntd.0007269.s004.pdf]
